# An uncharacterized region within the N-terminus of mouse TMC1 precludes trafficking to plasma membrane in a heterologous cell line

**DOI:** 10.1038/s41598-019-51336-0

**Published:** 2019-10-24

**Authors:** D. C. Soler, M. Manikandan, S. R. Gopal, A. E. Sloan, T. S. McCormick, R. Stepanyan

**Affiliations:** 1 The Department of Neurosurgery, University Hospitals Cleveland Medical Center, Cleveland, OH USA; 2 Brain Tumor and Neuro-Oncology Center, University Hospitals Cleveland Medical Center, Cleveland, OH USA; 3 Case Comprehensive Cancer Center, Case Western Reserve University, Cleveland, OH USA; 4 Department of Otolaryngology – HNS, Case Western Reserve University, Cleveland, OH USA; 5 Department of Dermatology, Case Western Reserve University, Cleveland, OH USA; 6 Murdough Family Center for Psoriasis, Case Western Reserve University, Cleveland, OH USA; 7 Department of Neurosciences, Case Western Reserve University, Cleveland, OH USA

**Keywords:** Cell biology, Molecular biology

## Abstract

Mechanotransduction by hair cell stereocilia lies at the heart of sound detection in vertebrates. Considerable effort has been put forth to identify proteins that comprise the hair cell mechanotransduction apparatus. TMC1, a member of the transmembrane channel-like (TMC) family, was identified as a core protein of the mechanotransduction complex in hair cells. However, the inability of TMC1 to traffic through the endoplasmic reticulum in heterologous cellular systems has hindered efforts to characterize its function and fully identify its role in mechanotransduction. We developed a novel approach that allowed for the detection of uncharacterized protein regions, which preclude trafficking to the plasma membrane (PM) in heterologous cells. Tagging N-terminal fragments of TMC1 with Aquaporin 3 (AQP3) and GFP fusion reporter, which intrinsically label PM in HEK293 cells, indicated that residues at the edges of amino acid sequence 138–168 invoke intracellular localization and/or degradation. This signal is able to preclude surface localization of PM protein AQP3 in HEK293 cells. Substitutions of the residues by alanine or serine corroborated that the information determining the intracellular retention is present within amino acid sequence 138–168 of TMC1 N-terminus. This novel signal may preclude the proper trafficking of TMC1 to the PM in heterologous cells.

## Introduction

The search for proteins forming hair cell mechano-electrical transduction (MET) ion channels has intensified over the last years. TMC1 and TMC2, the members of the transmembrane channel-like (TMC) family of proteins composed of eight isotypes, emerged recently as key MET channel components^1^. Specifically, several dominant and recessive mutations in human and murine TMC1 have been described to cause hearing loss and deafness^2–5^. TMC1 and TMC2 are expressed in the inner ear hair cells^2,5–7^ and are thought to have multiple transmembrane domains^7–10^, indicative of receptor, transporter, or ion channel function^2,8,11^. Furthermore, TMCs are related to the Anoctamin family of proteins, also known as TMEM16^8,10,12^, which are thought to have ten transmembrane domains and Ca^2+^-activated Cl^−^ channel activities^13–15^. mCherry-tagged TMC1 as well as AcGFP-tagged TMC2 localize predominantly to stereocilia tips in hair cells as they develop, and provide transgenic rescue of MET currents (both TMC1- and TMC2-based constructs) and hearing (TMC1-based construct) in double knockout *Tmc1*^*−/−*^; *Tmc2*^*−/−*^ mice^16^. Based on biochemistry, structural modeling, and hair-cell physiology approaches, studies suggest that TMC1 molecules assemble as a dimer and form the ion conduction pores of hair cell MET machinery^8,9^.

TMC1 and TMC2 are predicted to be membrane proteins^7^ and localize at the tips of the shorter rows of stereocilia^16,17^, the site of MET channel activity^18^. However, TMC1 and TMC2 expressed in heterologous cell lines consistently fail to localize to the plasma membrane (PM) and instead are retained in the endoplasmic reticulum (ER)^6,7,19^. The failure of TMC1 to traffic to the PM in heterologous cell lines is a major obstacle to studying the structure and function of TMC1. PM localization might also be indispensable for the assembly of the multiprotein complexes of TMC1 and other essential MET channel proteins for structural and functional analyses.

Although the lack of a tissue-specific chaperone in heterologous systems is thought to be responsible for the intracellular retention of TMC1^7^, it is also plausible that the chaperone interaction region within TMC1 is left unbound in heterologous cells, thus being instead recognized as an ER retention and/or degradation signal by the subcellular machinery. Since most ER retention signals are usually located at the N-terminus or C-terminus of membrane proteins^20^, we reasoned the existence of such a signal could be prohibiting localization of TMC1 to the PM in heterologous cells. However, precise identification of these signals can be an arduous task, requiring the use of radioactive labeling and technically complex methodologies accompanied by hard-to-interpret results^21,22^. Interestingly, even though TMC1 seems to contain known ER retention signals such as KK motifs within its N- and C-termini, their ablation by alanine substitutions does not improve trafficking^7^. Several canonical ER retention motifs such as KDEL or KKXX signals have been identified^20,23,24^, but many more may exist^21,25,26^, which bioinformatic analysis alone cannot predict^27^. Thus, their existence remains to be characterized and *de novo* ones could be present within TMC1 amino acid sequence.

Here, we report the development and application of a straightforward and robust approach that could shed light into the intractable nature of TMC1 trafficking problems. This novel method allows for identification of uncharacterized intracellular retention signals within the N- and C-termini of a given membrane protein of interest. We took advantage of the ability of Aquaporin 3-GFP (AQP3-GFP) to display intense PM labeling in heterologous systems with very low cytoplasmic fluorescence^28–30^. We then used this construct as a PM localization reporter to pinpoint possible novel intracellular retention signals located at TMC1 N- and C- termini that completely abrogate any detectable PM localization of AQP3-GFP. When we tagged the 183 amino acid N-terminus of TMC1 with AQP3-GFP, this construct displayed an ER-like localization pattern and absence of any detectable PM labeling. We identified that the N-terminal region between amino acids 138-168 (TMC1^138-168^) is responsible for intracellular retention and/or degradation. When tagged with AQP3-GFP, TMC1^138-168^ prevents any PM labeling; while the resulting pattern is reminiscent of TMC1 localization in heterologous cells. In addition, TMC1^138-168^ leads to a significant decrease in AQP3-GFP reporter intensity, suggesting that it may function as a degron. Substitution of TMC1^138-168^ with a scrambled peptide, containing the same amino acids but in random order, abolishes the ability of this fragment to preclude trafficking to the PM. Moreover, substitutions of amino acids at the edges of TMC1^138–168^ also resulted in robust PM labeling. Thus, TMC1^138–168^ does indeed contain a sequence-specific ability, which precludes trafficking to the PM.

## Results

### AQP3-GFP fusion protein displays unequivocally consistent membrane labeling in HEK293 cells

**Figure 1:**
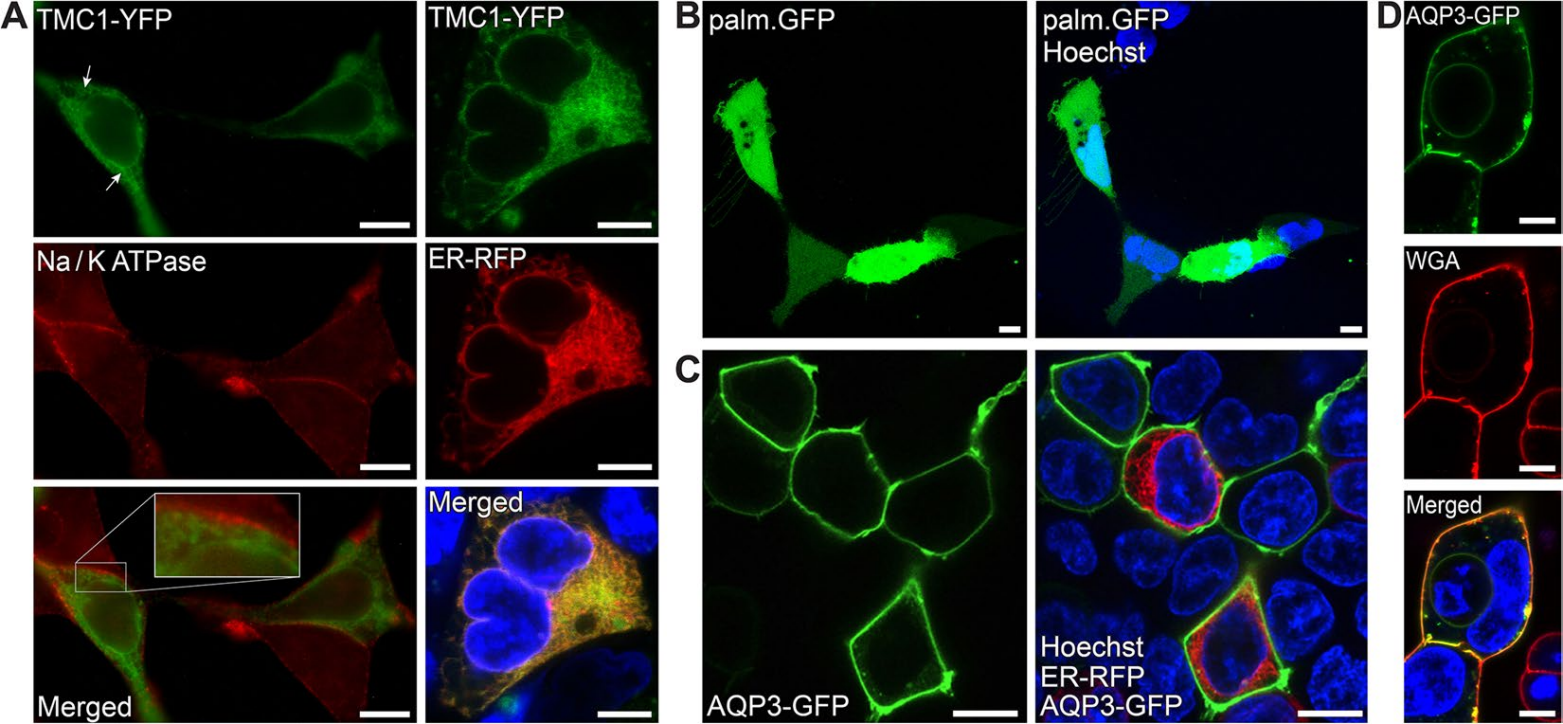
Heterologous expression of TMC1 and AQP3. (**A**) When expressed in HEK293 cells, TMC1 is retained in ER and no PM localization is observed (left column, top and bottom panels), when compared to membrane immunolabeling of Na/K ATPase (left column, middle panel). Co-labeling with ER-Tracker (right column) confirms predominant ER localization of TMC1-YFP in live HEK293 cells. (**B**) Palmitoylated-GFP displays both intense cytoplasmic and membrane localization. (**C**) Expression of AQP3-GFP in HEK293 cells produces unequivocal strong membrane labeling. (**D**) Co-labeling with wheat germ agglutinin (WGA) conjugate demonstrates prevalent membrane localization of AQP3-GFP in live HEK293 cells. Scale bars: 8 µm.

TMC1 is unable to properly traffic to the PM in heterologous systems when compared to other PM proteins such as Na/K ATPase (Fig. 1A). TMC1 gets invariably trapped at the ER in heterologous systems and never reaches the plasma membrane (Fig. 1A right panels). To identify the TMC1 regions which are responsible for precluding trafficking to the PM, we searched for a membrane protein with an available luminal C-terminus that could demonstrate strong PM labeling when fused with GFP. We first assessed a palmitoylated version of GFP, which has the ability to be anchored to the cell membrane in order to achieve PM labeling, as previously reported^31^. However, the cell cytoplasm is also stained intensely, impeding any clarity in the localization pattern (Fig. 1B). In addition, we considered the usage of a farnesylated version of EGFP, EGFP-F^32^, but farnesyltransferases require an unoccupied C-terminus^33^, thus are not suited for this study. We then opted for Aquaporin-3 (AQP3), an innocuous obliged tetrameric polytopic membrane protein able to transport water as well as glycerol and other solutes across cell membranes^34^. AQP3-GFP fusion produces an intensely bright PM labeling in HEK293 cells with minimal residual cytoplasmic fluorescence (Fig. 1C,D), when compared to labeling using other membrane proteins such as the Na/K ATPase (Fig. 1A) or CD8^35^. Importantly, the C-terminus of GFP is left free at the luminal side. We used this AQP3-GFP construct as a reporter to investigate the inability of TMC1 to traffic to the PM in heterologous cells.

### The 183 amino acid N-terminus of mouse TMC1 precludes AQP3-GFP trafficking to the plasma membrane

**Figure 2:**
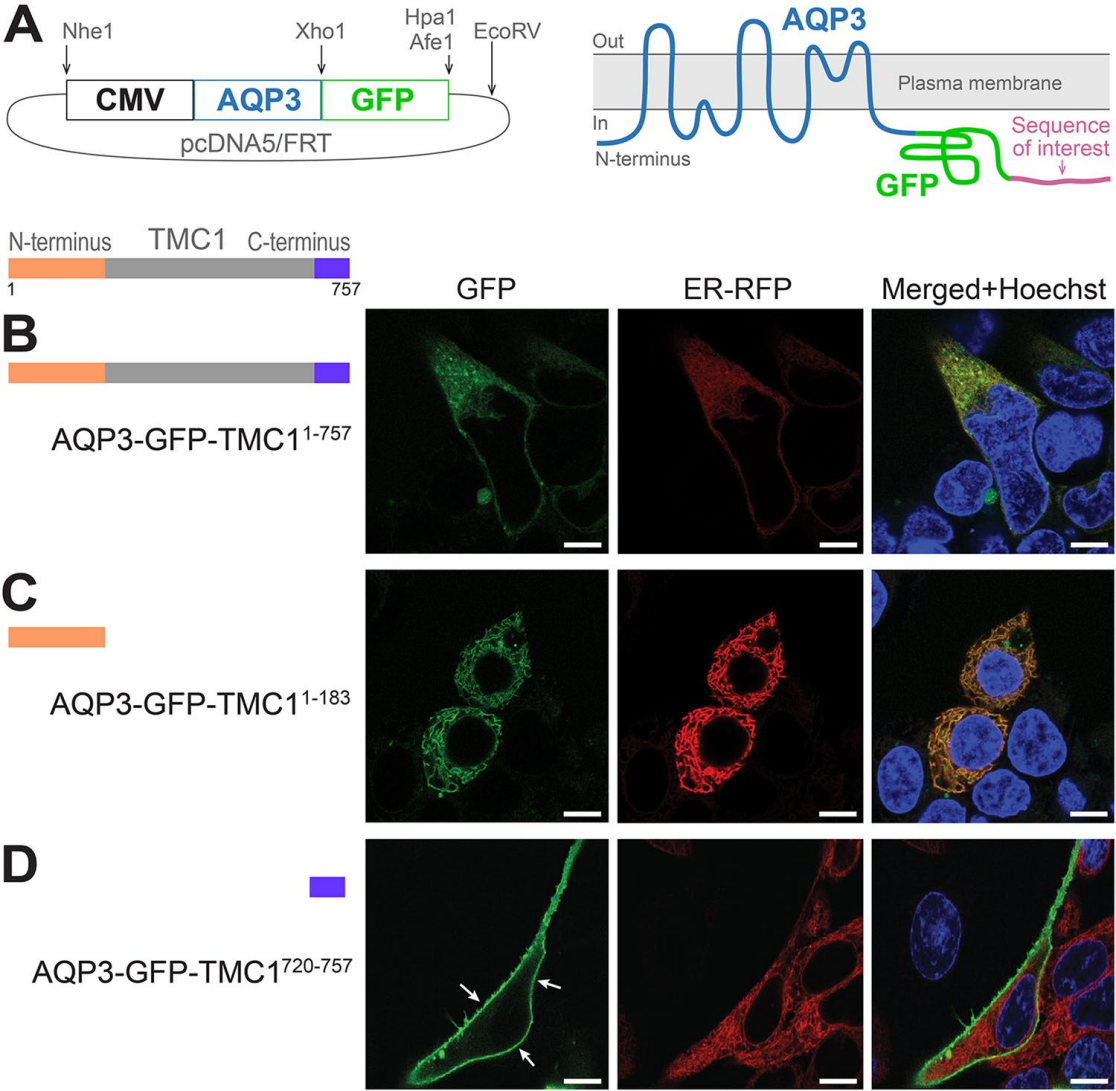
AQP3-GFP reports intracellular retention quality of TMC1. (**A**) Schematic representation of AQP3-GFP reporter construct. A protein sequence of interest could be fused to the C-terminus of AQP3-GFP and tested for its ability to prevent AQP3-GFP from trafficking to the PM. (**B**) When fused to the C-terminus of AQP3-GFP, expression of full-length TMC1 results in an ER-like labeling pattern in HEK293 cells. (**C**) When only the N-terminus of TMC1 was tagged with AQP3-GFP, an ER-like labeling pattern was observed as well, without any noticeable PM fluorescence. (**D**) C-terminus of TMC1, when fused to AQP-GFP, does not preclude PM localization of the reporter (arrows). Scale bars: 8 µm.

We first tested whether heterologously expressed full murine TMC1, fused to the C-terminal of AQP3-GFP, could successfully traffic to the PM (Fig. 2A). We substituted residues of potential ER canonical retention signals of TMC1 with alanines in this and all subsequent constructs we tested. The AQP3-GFP-TMC1 displayed an ER-like localization pattern when expressed in HEK293 cells, similar to TMC1-YFP labeling (Figs 2B and 1A respectively). Thus, replicating the reticular pattern of TMC1, AQP3-GFP construct is suitable to identify regions responsible for halting trafficking to the PM.

To test whether the N-terminus of TMC1 (TMC1^1-183^) contains such a region, TMC1^1-183^ was fused to the C-terminus of AQP3-GFP. We estimated the length of TMC1 N- and C-termini based on the transmembrane prediction tool from the Department of Bio and Health informatics in the Center for Biological Sequence Analysis from the Technical University of Denmark. The generated prediction is in agreement with the most recently proposed topology of TMC1^8,9^. When expressed in HEK293 cells, AQP3-GFP-TMC1^1-183^ labeling resembles the intracellular reticular pattern of AQP3-GFP-TMC1 and TMC1-YFP constructs (Figs 2C,B and 1A respectively). This suggests that, at least the N-terminus of TMC1, contains an uncharacterized region which prevent AQP3-GFP and presumably TMC1 from reaching the PM in heterologous systems. Next, we tested the C-terminus of TMC1 (TMC1^720-757^). AQP3-GFP-TMC1^720-757^ managed to reach the PM (Fig. 2D), suggesting the C-terminus of TMC1 does not preclude trafficking to the PM.

### The N-terminal region of TMC1, encompassing amino acids 138-168, precludes trafficking to the plasma membrane

**Figure 3:**
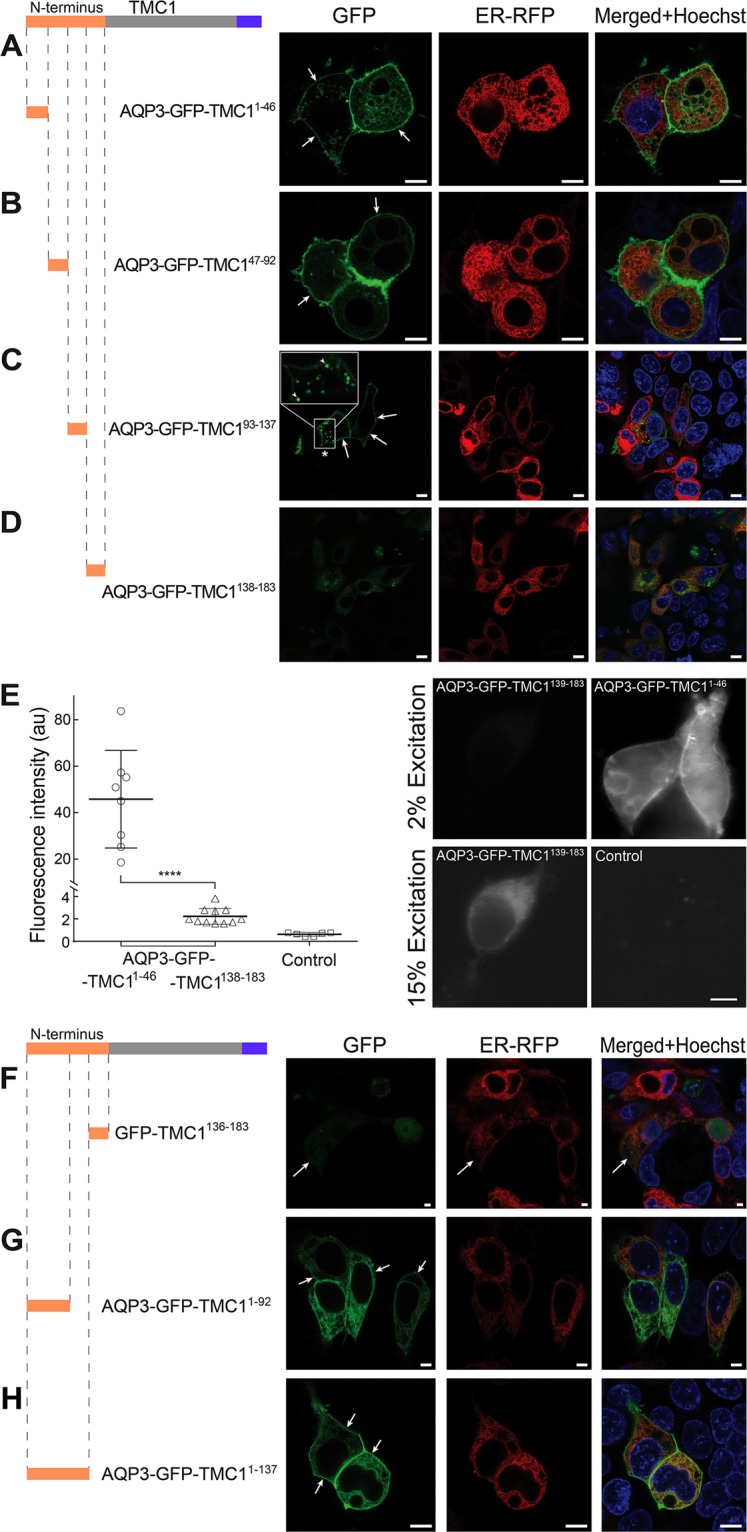
Discerning the region within TMC1 N-terminus that precludes reporter trafficking to the PM in HEK293 cells. (**A**) N-terminal region of TMC1 composed of amino acids (aa) 1-46 (TMC1^1-46^) does not preclude trafficking of AQP3-GFP to the PM (top row, white arrows). (**B**,**C**) TMC1 N-terminal aa sequences 47-92 (B, white arrows) and 93-137 (C, white arrows) do not preclude trafficking of the reporter to the PM. (**D**) TMC1 N-terminal aa sequence 138-183 (TMC1^138-183^) prevents AQP3-GFP from reaching the PM with a concomitant decrease in reporter fluorescence intensity (intensity of green channel is enhanced). (**E**) Quantification of AQP3-GFP-TMC1^138-183^ signal intensity as compared to AQP3-GFP-TMC1^1-46^. When tagged with AQP3-GFP, TMC1^138-183^ induces a substantial decrease in reporter intensity. Fluorescent signal of AQP3-GFP-TMC1^138-183^ only becomes apparent if excitation light intensity is increased from 2% of maximal value (top micrograph) to 15% (bottom micrograph, the same field of view). ****p < 0.0001, ANOVA with Tukey post hoc test. Control – identically treated untransfected cells. (**F**) When tagged along with GFP alone, the TMC1^138-183^ fragment reduces the signal intensity and reproduces a reticular intracellular pattern (white arrow). (**G**,**H**) When tagged with AQP3-GFP, first half of TMC1 N-terminus, TMC1^1-92^ (**G**), and fragment containing two thirds of N-terminus, TMC1^1-137^ (**H**), were able to reach the PM (white arrows), although a strong ER pattern was evident. Scale bars: 8 µm (**A**–**D**,**G**,**H**); 5 µm (**E**); 3 µm (**F**).

To narrow down the region within the N-terminus of TMC1 which halts trafficking to the PM, four constructs, each containing consecutive sequences of N-terminus, TMC1^1-46^, TMC1^47-92^, TMC1^93-137^, and TMC1^138-183^, were fused to the C-terminus of AQP3-GFP. When expressed in HEK293 cells, AQP3-GFP-TMC1^1-46^ and AQP3-GFP-TMC1^47-92^ constructs predominantly localized to the PM with some weak reticular pattern (Fig. 3A,B). This indicates that the first half of the N-terminus of TMC1 does not preclude trafficking to the PM. AQP3-GFP-TMC1^93-137^ trafficked to the PM as well, although a distinct intracellular localization pattern was also noticed (Fig. 3C, asterisk). Next, AQP3-GFP-TMC1^138-183^ was expressed in HEK293 cells. As a result, an unexpected drop in reporter intensity was observed, compared to the other three TMC1 N-terminal fragment containing constructs. This drop in expression was accompanied by a complete absence of PM localization (Fig. 3D). Thus, these results suggest that the fourth quarter of TMC1 N-terminus (amino acid residues 138-183) precludes membrane protein AQP3 from trafficking to the PM in HEK293 cells. Moreover, the ability of this region to significantly reduce the intensity of AQP3-GFP or just GFP (Fig. 3D–F), is suggestive of a “degron” signal presence within TMC1^138-183^. To test whether any putative intracellular retention signals were perturbed by splitting TMC1 N-terminus into four parts, we generated two larger constructs containing the split regions, TMC1^1-92^ and TMC1^1-137^. These fragments, containing approximately half and three quarters of the N-terminus of TMC1 respectively, were tagged with AQP3-GFP: AQP3-GFP-TMC1^1-92^ and AQP3-GFP-TMC1^1-137^. When expressed in HEK293 cells, TMC1^1-92^ and TMC1^1-137^ did not prevent the AQP3-GFP from reaching the PM, but did display significant reticular localization patterns as well (Fig. 3G,H respectively).

**Figure 4:**
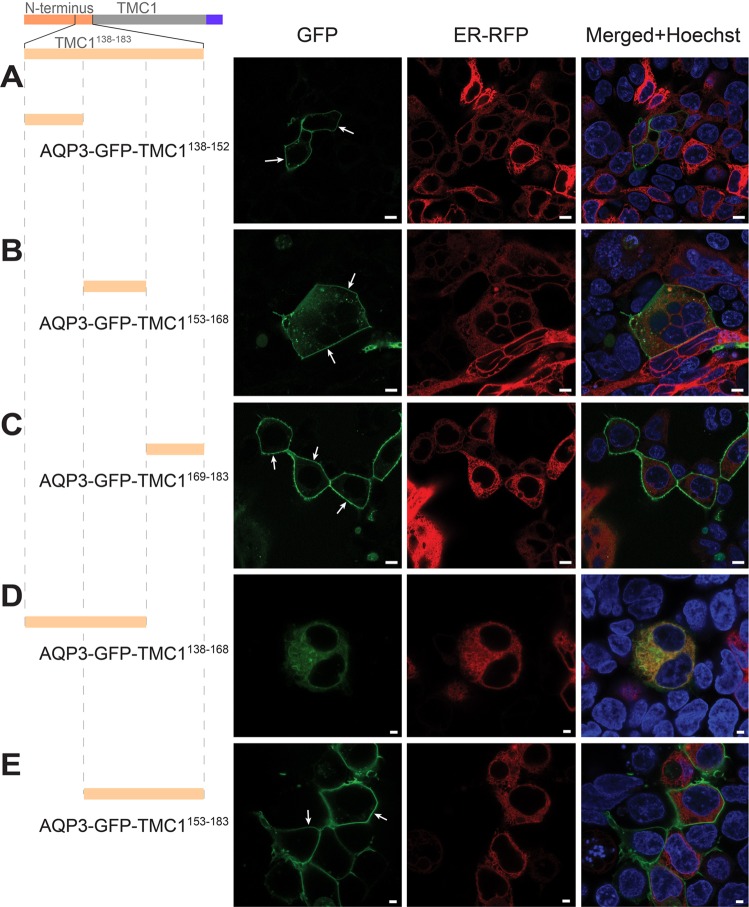
TMC1 N-terminus region between amino acids 138-168 precludes trafficking to the PM. (**A**–**C**). Fragments TMC1^138-152^, TMC1^153-168^, and TMC1^169-183^ are able to reach the PM, when tagged with AQP3-GFP. (**D**) Fragment TMC1^138-168^, when tagged with AQP3-GFP, is sufficient to cause ER localization pattern and a decrease in reporter intensity. (**E**) When the overlapping 31 amino acids TMC1^153-183^ are tagged to AQP3-GFP, membrane localization and absence of ER staining are evident (white arrows). Scale bars: 8 µm (**A**–**C**); 3 µm (**D**,**E**).

Further, we aimed to narrow down the localization of the region residing within TMC1^138-183^ that prevents trafficking to the PM. Hence, we tested three consecutive parts of the TMC1^138-183^ region: TMC1^138-152^, TMC1^153-168^, and TMC1^169-183^. Interestingly, all of these three regions, when tagged with AQP3-GFP, reached the PM (Fig. 4A–C). We then tested two additional overlapping segments, TMC1^138-168^ and TMC1^153-183^. When TMC1^138-168^ was tagged with AQP3-GFP, it produced a significant reduction in reporter intensity (Fig. 4D, compared to those constructs described in Fig. 3D–F). Moreover, we did not observe any PM localization of AQP3-GFP-TMC1^138-168^, which is reminiscent of AQP3-GFP-TMC1^138-183^ localization. In contrast, AQP3-GFP-TMC1^153-183^ did reach the PM (Fig. 4E) without any decrease in reporter intensity levels. This suggests that, specifically, the region within amino acid residues 138-168 of TMC1 N-terminus precludes trafficking to the PM. TMC1^138-168^ corresponds to AFKMMMA*AA*WAKFLRDFENFKAACVPWENKI (notice: italic AA substitutes potential ER canonical retention signal KK in the original sequence).

**Figure 5:**
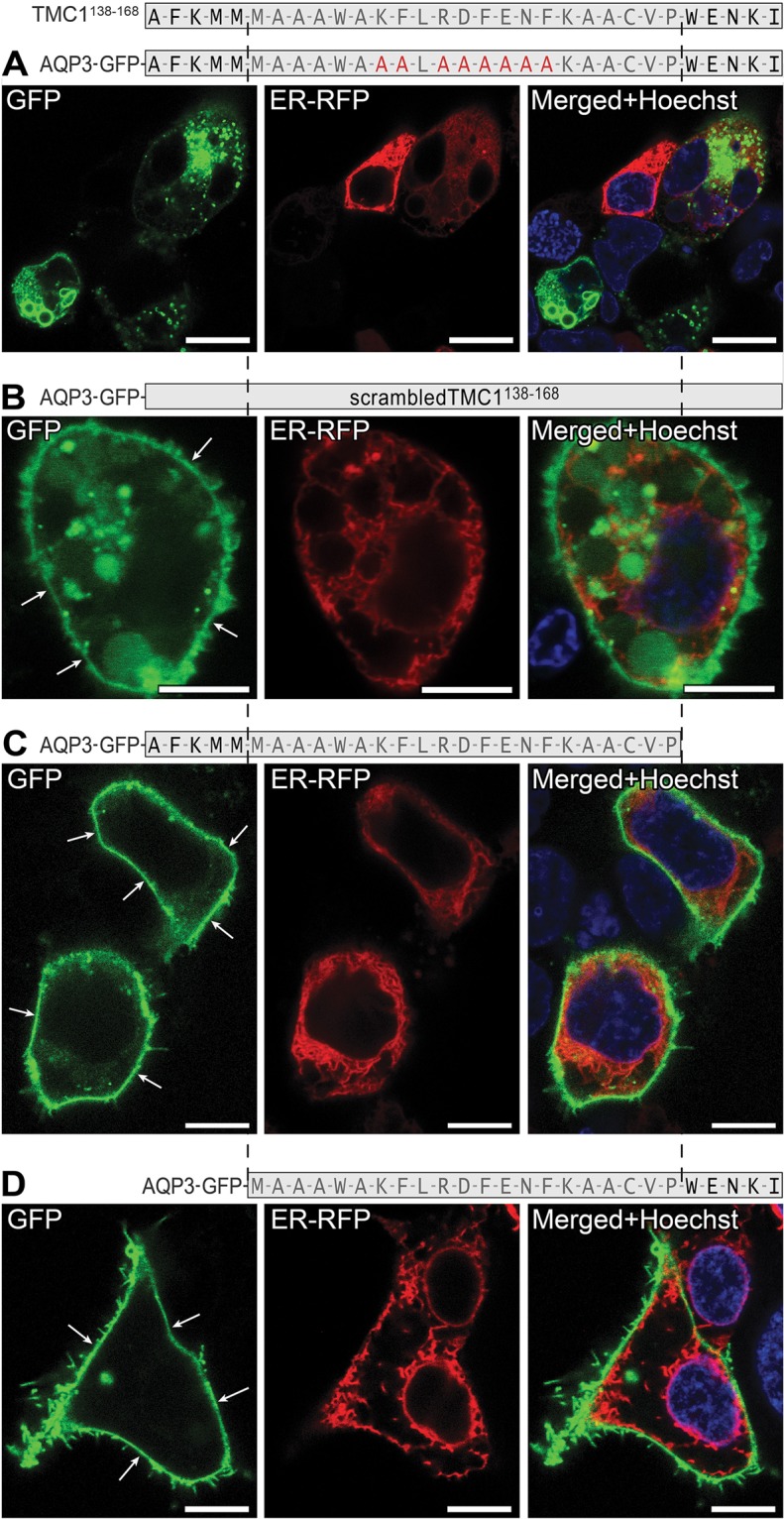
Amino acid residues at either ends of TMC1^138-168^ are critical for its activity. (**A**) Multiple alanine substitutions within the middle segment of TMC1^138-168^ failed to affect the ability of this region to prevent reporter trafficking to the PM. (**B**) A scrambled peptide of TMC1^138-168^ fragment is not able to prevent AQP3-GFP trafficking to the PM. (**C**,**D**) When residues WENKI (**C**) or AFKMM (**D**) at the ends of TMC1^138-168^ are removed, the resulting TMC1^138-163^ (**C**) or TMC1^142-168^ (**D**) fragments were able to reach the PM when tagged with AQP3-GFP. Scale bars: 3 µm.

Since constructs TMC1^138-152^ and TMC1^153-168^ produced PM localization when tagged to AQP3-GFP reporter (Fig. 4A,B), we hypothesized that the region around the split, residues 152-153, is responsible for the intracellular retention. To test this, we substituted residues 149K, 150F, 152R, 153D, 154F, 155E, 156N, and 157F with alanine. However, such substitutions did not alter the intracellular retention activity of TMC1^138-168^ (Fig. 5A). Further, we tested TMC1^142-168^ and TMC1^138-163^ (Fig. 5C,D). Neither shortened region precluded the reporter from reaching the PM, suggesting that amino acid residues at either end, but not in the middle of the TMC1^138-168^ sequence, are critical in precluding trafficking to PM. Interestingly, a scrambled peptide containing the same amino acids as in TMC1^138-168^ but in random order does not preclude AQP3-GFP from reaching the PM (Fig. 5B), suggesting that the activity of the TMC1^138-168^ region is sequence-specific.

### TMC1 N-terminus with introduced mutations within TMC1^138-168^ does not preclude trafficking to the plasma membrane

**Figure 6:**
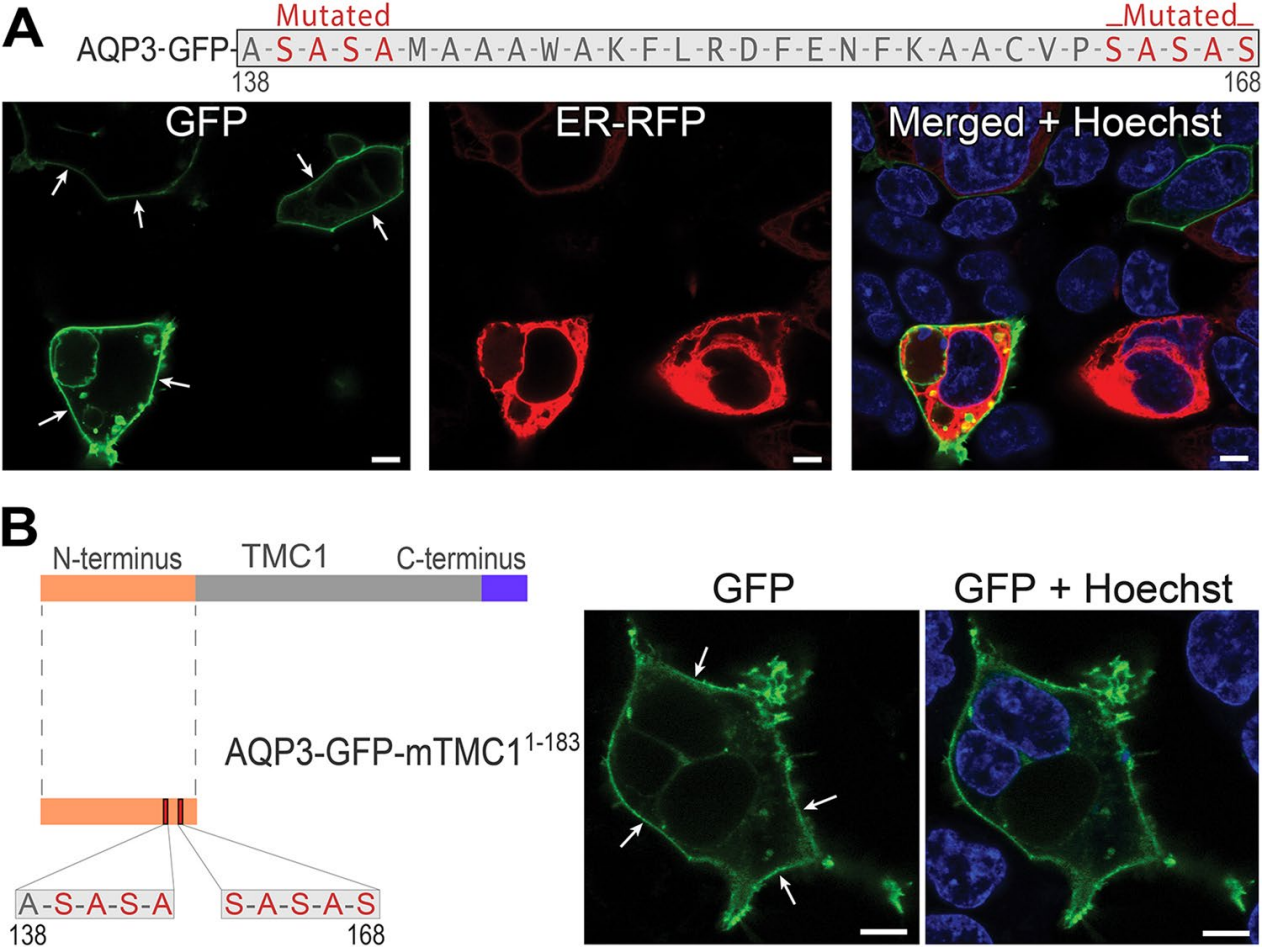
Alanine and serine substitutions at the ends of TMC1^136-168^ sequence invoke N-terminus of TMC1 trafficking to the PM. (**A**) When key residues AFKMM and WENKI at either ends of TMC1^138-168^ are substituted by alanine and serine, the resulting mutant TMC1^138-168^ (mTMC1^138-168^) is able to reach the PM when tagged with AQP3-GFP. (**B**) Likewise, reporter tagged mutant full N-terminus of TMC1 (mTMC1^138-168^), harboring AFKMM and WENKI substitutions, is able to traffic to the PM. Scale bars: 8 µm

To investigate the ability of the TMC1^138-168^ region to preclude trafficking to PM, we used alanine and serine mutagenesis to substitute both ends of the TMC1^138-168^ sequence, AFKMM and WENKI amino acids, with ASASA and SASAS. When tagged with AQP3-GFP reporter, mutant TMC1^138-168^ (mTMC1^138-168^) reached the PM (Fig. 6A). We then introduced the same mutations within the entire N-terminus of TMC1. When tagged with AQP3-GFP, the mutant N-terminus of TMC1, containing the alanine- and serine-substituted AFKMM and WENKI residues, AQP3-GFP-mTMC1^1-183^, was able to traffic to the PM of HEK293 cells (Fig. 6B). This is in stark contrast to wild type N-terminus, which precludes reporter trafficking to the PM (Fig. 2C).

## Discussion

Members of the transmembrane channel-like family, TMC1 and TMC2, are key proteins in hair cell mechanotransduction, which enables hearing in vertebrates. TMCs are thought to form the permeation pathways of MET ion channel complexes^8,9^. Inability of TMCs to traffic to the PM in heterologous cell systems is a major obstacle to studying their structure and function. Although significant progress has been recently achieved, indicating TMC1 might form a dimer with predicted ion channel properties^8^, the trafficking issue is still a significant barrier impeding further work on the physiology and structure of the MET apparatus. AQP3-GFP-based reporter might provide a promising new tool to uncover protein regions, which preclude trafficking to the PM. When expressed in HEK293 cells, AQP3-GFP labels PM with unequivocal intense staining (Fig. 1C,D) and, importantly, with minimal residual cytoplasmic localization. When TMC1 is fused to the C-terminus of AQP3-GFP, the characteristic intracellular localization of TMC1 in HEK293 cells is not disrupted (Fig. 2B). This allowed us to use AQP3-GFP as a PM reporter in order to query TMC1 for regions, which preclude any detectable trafficking to the PM.

In this study, using HEK293 cells, we heterologously expressed the full and partial sequences of TMC1, tagged with AQP3-GFP. The heterologous expression revealed that the N-terminus of TMC1 and not the C-terminus, contains a region that prevents any detectable trafficking of AQP3-GFP reporter to the PM (Figs 2C,D, 3D–F and 4D). The sequence is positioned within amino acids 138-168, since expression of amino acids 138-152 or 153-168 of TMC1, when tagged with AQP3-GFP, reach the PM (Fig. 5). Moreover deletion of the five amino acids at either end of TMC1^138-168^, AFKMM or WENKI, abolish its ability to prevent trafficking to the PM (Fig. 5). Interestingly, when residues AFKMM and WENKI were substituted for ASAS and SASAS respectively, mTMC1^138-168^ was able to reach the PM, as well as mTMC^1-183^ (full N-terminus) (Fig. 6). TMC1^138-168^ region also contains uncharacterized degron activity, which significantly decreases expression of AQP3-GFP, as well as soluble GFP (Fig. 3D–F). We further characterized TMC1^138-168^ and found that it may contain a split signal composed of residues involving AFKMM and WENKI, both of which must be present to completely abrogate PM localization of AQP3-GFP reporter (Fig. 5C,D).

Nevertheless, TMC1 without 183 amino acid N-terminus still failed to reach the PM, when tagged with AQP3-GFP (data not shown). This result suggests that either lack of N-terminus results in a misfolded protein or additional ER retention motifs might still be present outside N-terminus. Since TMC1 has the ability to traffic to the PM at the tips of stereocilia, it is possible the ER retention signals that trap TMC1 in heterologous cells are masked by the action of specific protein partners in hair cells.

Pure bioinformatical analysis of TMC1 might be unable to pinpoint the existence of uncharacterized ER motifs or any other regions responsible for halting trafficking to the PM^27^. Here we aimed to shed light into the possible signals, present within the N- and C-termini of TMC1, which may prevent TMC1 from trafficking to the PM in heterologous cell lines. Our data explains, at least partly, why TMC1 is retained intracellularly and fails to successfully traffic to the PM. AQP3-GFP could be further used as a PM reporter to examine putative transmembrane domains as well as inner and outer loops of TMC1 that could contain additional uncharacterized regions, which preclude trafficking to the PM. AQP3-GFP-based constructs could also be employed to study other membrane proteins that do not traffic to PM when expressed heterologously.

In conclusion, we have successfully applied AQP3-GFP-based Reporter (termed AGR) to effectively identify a hitherto uncharacterized region, which may contribute to the lack of PM localization of TMC1 in heterologous cell systems.

## Materials and Methods

### Plasmid constructs

The sequence of turboGFP variant (Evrogen, Moscow, Russia) was fused to the C-terminal of human AQP3 (UniProtKB/Swiss-Prot #Q92482.2) *in silico* using Lasergene II software (DNASTAR, Madison, WI). The full cDNA containing AQP3-GFP was then synthesized by Genscript (Piscatawas, NJ). The cDNA was subcloned into a pcDNA5/FRT plasmid (Invitrogen, Carlsbad, CA) using restriction enzymes Hind3/EcoRV. Each murine TMC1 (Pubmed #NP_083229.1) construct was also based on custom synthesis by Genscript with its known ER retention motifs KK or RRR substituted for alanines, and codon optimized for mammalian expression in HEK293 cells. Each *Tmc1* based sequence was then subcloned into the AQP3-GFP-pcDNA5/FRT plasmid using Hpa1/EcoRV restriction sites. The palmitoylated GFP was custom synthesized, codon optimized for mammalian HEK293 expression and subcloned into pcDNA5/FRT plasmid using Nhe1/EcoRV restriction sites by Genscript. All constructs were confirmed by sequencing.

To express TMC1-YFP construct, the C-terminus of coding region of mouse *Tmc1* was fused to the coding sequence of mVenus reporter using Hind III and BamHI sites in an mVenus-N1 vector^36^. Sequencing of this construct confirmed the identity as murine *Tmc1* (Pumbed NM_028953).

### Cell culture, transfection

HEK293 cells were purchased from Life Sciences (Carlsbad CA) and seeded in 50 mm glass-bottom dishes (WPI, Sarasota, FL) or 12-well glass-bottom culture plates (Cellvis, Mountain View, CA) and cultured in DMEM (Life Technologies, Carlsbad, CA) supplemented with 10% Fetal Bovine Serum and 1% pen/strep for 3 days prior to transfection. On the day of transfection, spent media was replaced by fresh media and plasmid constructs were transfected into HEK293 cells using Lipofectamine 2000 (Life Technologies, Carlsbad, CA) or K2 Transfection System (Biontex, Munchen, Germany) following the manufacturer’s instructions. Red Fluorescent Protein based BacMam 2.0 constructs specific for ER were co-transfected the same day following manufacturer’s instructions (Thermo Fisher Scientific, Waltham, MA). ER-Tracker™ Red (Thermo Fisher Scientific) was used for live cell imaging as well (Fig. 1A only). To mark the plasma membrane in live cells, we used wheat germ agglutinin (WGA) CF594 conjugate (Biotium, Fremont, CA). To label the cell nucleus we used Hoechst (Life Technologies, CA). All recorded experiments were performed 48 h after transfection. The aim of the experiments was to detect a region that precludes any detectable PM labeling, therefore we used the following criteria: was PM localization detected or was PM labeling absent in all three independent experiments.

### Immunocytochemistry

HEK293 cells were fixed in 1% paraformaldehyde in PBS for 30 minutes at room temperature, permeabilized with 100% methanol for 5 minutes at −20 °C, and washed with PBS 3 times, 5 minutes each. Then, HEK293 cells were blocked with 10% normal goat serum and 1% BSA in PBS for 30 minutes at room temperature and incubated with the mouse polyclonal Na/K ATPase antibody (α5, Developmental Studies Hybridoma Bank at University of Iowa, Iowa City, IA; deposited by Fambrough, D.M.) in the blocking buffer (antibody dilution 1:200) for 1 hour at room temperature. Cells then were washed 3 times with PBS for 5 minutes. Specific labelling of Na/K ATPase antibody was visualized with secondary goat anti-mouse antibody conjugated to Alexa Fluor 594 (1:500 dilution; Jackson ImmunoResearch Laboratories, Inc., item # 115-585-146). HEK293 were incubated in secondary antibody for an hour, followed by 3 washes of 5 minutes each, with PBS at room temperature.

### Protein topology

Protein topology was guided by using the predictive online tool http://www.cbs.dtu.dk/services/TMHMM/ from the Department of Bio and Health informatics in the Center for Biological Sequence Analysis from Technical University of Denmark.

### Imaging

Transfected HEK293 cells were examined with an SP8 confocal fluorescence microscope using 63 × 1.4 NA objective, Leica (Wetzlar, Germany). Alternatively, cells were observed microscopically using an upright Olympus BX51WI equipped with 100 × 1 NA objective and images were captured with a Grasshopper3 CMOS camera (FLIR, Richmond, BC, Canada) controlled by manufacturer provided software. To generate images for quantitative analysis, we kept camera, microscope, and excitation light source (X-Cite 120LED*Boost*, Excelitas Technologies, 2% intensity) settings constant. To observe cells with low-level fluorescence, excitation light was set at 15% of intensity. Fluorescence intensities were obtained using Fiji^37^. Regions of interest were used to obtain average intensity values from the transfected HEK293 cells (I_cell_) and an area without cells (I_background_) within the same image. Fluorescence intensity of transfected HEK293 cells (I_norm_) for each cell was normalized (I_norm_ = I_cell_ − I_background_). Each data sample represent average normalized intensity of the transfected cells within an image. We captured 3 to 6 fields of view per dish, using two dishes per construct in every experiment.

### Statistics

All statistical analyses were performed using GraphPad Prism 7. Data are reported as mean ± SEM. Comparisons between groups were analyzed by ANOVA with Tukey post hoc testing.
